# Contrasting Patterns in Mammal–Bacteria Coevolution: *Bartonella* and *Leptospira* in Bats and Rodents

**DOI:** 10.1371/journal.pntd.0002738

**Published:** 2014-03-20

**Authors:** Bonnie R. Lei, Kevin J. Olival

**Affiliations:** 1 EcoHealth Alliance, New York, New York, United States of America; 2 Department of Organismic and Evolutionary Biology, Harvard University, Cambridge, Massachusetts, United States of America; University of Tennessee, United States of America

## Abstract

**Background:**

Emerging bacterial zoonoses in bats and rodents remain relatively understudied. We conduct the first comparative host–pathogen coevolutionary analyses of bacterial pathogens in these hosts, using *Bartonella* spp. and *Leptospira* spp. as a model.

**Methodology/Principal Findings:**

We used published genetic data for 51 *Bartonella* genotypes from 24 bat species, 129 *Bartonella* from 38 rodents, and 26 *Leptospira* from 20 bats. We generated maximum likelihood and Bayesian phylogenies for hosts and bacteria, and tested for coevoutionary congruence using programs ParaFit, PACO, and Jane. *Bartonella* spp. and their bat hosts had a significant coevolutionary fit (ParaFitGlobal = 1.9703, P≤0.001; m^2^ global value = 7.3320, P≤0.0001). *Bartonella* spp. and rodent hosts also indicated strong overall patterns of cospeciation (ParaFitGlobal = 102.4409, P≤0.001; m^2^ global value = 86.532, P≤0.0001). In contrast, we were unable to reject independence of speciation events in *Leptospira* and bats (ParaFitGlobal = 0.0042, P = 0.84; m^2^ global value = 4.6310, P = 0.5629). Separate analyses of New World and Old World data subsets yielded results congruent with analysis from entire datasets. We also conducted event-based cophylogeny analyses to reconstruct likely evolutionary histories for each group of pathogens and hosts. *Leptospira* and bats had the greatest number of host switches per parasite (0.731), while *Bartonella* and rodents had the fewest (0.264).

**Conclusions/Significance:**

In both bat and rodent hosts, *Bartonella* exhibits significant coevolution with minimal host switching, while *Leptospira* in bats lacks evolutionary congruence with its host and has high number of host switches. Reasons underlying these variable coevolutionary patterns in host range are likely due to differences in disease-specific transmission and host ecology. Understanding the coevolutionary patterns and frequency of host-switching events between bacterial pathogens and their hosts will allow better prediction of spillover between mammal reservoirs, and ultimately to humans.

## Author Summary

Bats and rodents are important hosts for emerging human diseases. While a large body of research has focused on viral pathogens in these hosts, the diversity, evolution, and transmission of their bacterial pathogens remains relatively unstudied. We conducted co-evolutionary analyses of two bacterial genera know to be pathogenic in humans, *Bartonella* and *Leptospira*, along with their bat and rodent hosts. We found that *Bartonella* had a significant pattern of coevolution with both bat and rodent hosts, while *Leptospira* in bats showed a lack of congruence with its bat hosts and a high number of host switching events. Our statistically driven approach to understand the frequency of host switching events in these mammal–bacterial systems can be easily applied to other host–pathogen systems, including viruses, to assess the likelihood of zoonotic spillover.

## Introduction

Bats and rodents are the two most diverse and geographically widespread orders of mammals [1], [2], and are important reservoirs for a growing number of emerging infectious diseases (EIDs) with significant impacts on public health. Bats are reservoir hosts of several viral pathogens of high consequence, including Henipaviruses, Ebola and Marburg viruses, lyssaviruses, Severe Acute Respiratory Syndrome coronavirus, and likely Middle Eastern Respiratory Syndrome coronavirus [3]–[5]. Rodents are known reservoirs of hantaviruses, arenaviruses, Lassa fever virus, plague and other bacterial zoonoses [6]. Over the last two decades, the majority of research on bat and rodent zoonotic diseases has focused on viral infections (Figure S1). While the number of virus-related publications for bats has had a marked rise over the past decade, research on bacteria in bats has remained consistently low (Figure S1). The evolutionary relationships between these important mammalian hosts and their known bacterial pathogens has been little studied to date [7], [8].

Bats and rodents are evolutionarily ancient orders of mammals, with periods of diversification dating back 75 and 85 million years ago, respectively, thus allowing ample time for pathogens and hosts to coevolve [9]. Bats and rodents make up 60% of all extant mammal species while exhibiting a wide-range of life-history and ecological traits. Ecological, evolutionary, and life-history traits can influence pathogen richness and cross species transmission, or spillover, in these bat and rodent hosts [5], [8]–[11]. The peridomestic habits of these mammals also likely increase the frequency of human contact and facilitate disease spillover [12], [13]. Anthropogenic alterations that increase exposure to bats and rodents, including expanding agricultural operations, bushmeat hunting, and climate change, may increase the opportunity for diseases to emerge in human populations in the future [14]. How these ecological and life history factors may affect the coevolutionary patterns between reservoir host species and their associated pathogens is an open question, but will depend on characteristics related to pathogen transmission and host ecology.

The evolutionary patterns of hosts and their known pathogens can be used to quantify the frequency of spillover events within and between reservoir hosts, and is a crucial first step for developing predictive models for zoonotic disease emergence. Previous research has demonstrated how these coevolutionary studies can shed light on specific instances of host switching, cospeciation, and other events in coronaviruses and their bat hosts [15], as well as malaria parasites and their avian hosts [16]. However, to our knowledge, no comparative cophylogenetic analysis of bacterial pathogens has been applied yet to bat and rodent hosts. Here we examine host-pathogen evolution in bats and rodents using two bacterial genera, *Bartonella spp.* and *Leptospira spp.*, known to cause neglected tropical diseases in humans.

The genus *Bartonella* consists of globally distributed and highly diverse alpha-proteobacteria that infects a wide-range of mammals. After infection, the bacteria eventually enters erythrocytes and endothelial cells and can persist asymptomatically in a wide range of mammalian reservoir hosts [17]. The disease is mainly transmitted through arthropod vectors including fleas, flies, lice, mites, and ticks [18]–[21]. Thus, the transmission and evolution of *Bartonella* species in mammals is the result of a complex relationship between multiple hosts, vectors, and pathogens. *Bartonella* has been reported with high prevalence and genetic diversity from numerous recent studies in bats [22]–[25] and rodents [26]–[30]. *Bartonella* is recognized as a neglected tropical disease, and there are indications of human infections derived from neighboring wildlife populations. In Thailand, genetic studies have indicated highly similar *Bartonella* strains between infected humans and nearby rodent populations [31]–[34]. Neighboring rodents have also been implicated as a possible source for bartonellosis in the United States and Nigeria [35], [36].

*Leptospira* is a genus of spirochete bacteria which also has a wide geographical distribution [37], and has been recognized as an important emerging pathogen due to its increasing incidence in both developing and developed countries [38]. Leptospires are maintained in nature by a large variety of wild and domestic animal hosts, and the bacteria colonize their kidneys and are excreted in their urine [39]. Rodents were the first recognized carriers, though the bacteria has been isolated from almost all screened mammals. Recently, bats have been found to carry *Leptospira* in Madagascar, Australia, Peru, and Brazil, and seroprevalence has been recorded to be as high as 35% [40]–[44]. Unlike *Bartonella* spp., *Leptospira* spp. are not vector-borne, and transmission to humans and other hosts is primarily through contact with water and environments contaminated with infected animal urine [45]. While most research has focused on rodents reservoirs of leptospirosis, recent genetic studies have also indicated bats as carriers of the bacteria [41], [44].

In order to better understand the evolutionary dynamics of *Bartonella* and *Leptospira* in bat and rodent hosts, we compiled available genetic information from hosts and bacterial pathogens to determine cophylogenetic patterns on a global scale. Evidence of cophylogeny can be used to test hypotheses of coevolution, and a lack of congruence between host and pathogen phylogenies can identify pathogen spillover, or interspecific transmission, events [46]. Long associations through evolutionary time can lead to reciprocal adaptations in both the hosts and their parasites, as well as concurrent divergence events in the two lineages. Evolutionary events including strict codivergence, parasite duplication, parasite extinction, and parasite host switching, will either strengthen or diminish the congruence between host and parasite [47]. Patterns of host-parasite or host-pathogen congruence may also vary geographically. For example, host specificity of *Bartonella* was observed in Old World bats in Kenya [24], while bats in Peru and Guatemala in the New World appeared to have no specific *Bartonella*-bat relationships [22], [48]. In contrast, consistency is observed for *Bartonella* in rodents, with host-specificity apparent in both Old and New World [49]–[51].

The primary goal of this paper is to examine the global co-evolutionary patterns of bats, rodents and their associated bacterial pathogens – using *Bartonella* and *Leptospira* as case studies. We specifically test for evolutionary congruence between bat host species and *Bartonella* and *Leptospira*, as well as rodent host species and *Bartonella*. Analysis of rodent *Leptospira* was unfortunately excluded due to a lack of comparable sequence datasets and host taxonomic diversity. Although there is a long history of research on leptospirosis in rodents, the publicly available sequence data that has been obtained thus far covers only a handful of rodent host species distributed across 3 genes: secY, flab,and lipL3 [52]–[56]. We also test whether evolutionary patterns and bacterial host specificity differ between the New World and Old World bat and rodent hosts, as was previously observed for *Bartonella* [22], [24], [48]. Finally, we conduct event-based cophylogeny analyses to reconstruct likely evolutionary histories for each group of pathogens and hosts.

## Materials and Methods

### Compiled sequence data

Sequence data used for analyses were obtained by searching for relevant papers from 1900–2013 through online sources PubMed, Web of Science, and Google Scholar using keywords “Bartonella*” and “Leptospir*” combined with “bat OR Chiroptera*” or “rodent*”. All *Bartonella* and *Leptospira* sequences from bat or rodent hosts identified to the species level were compiled into our datasets (Tables S1, S2, S3). Bat hosts include individuals in the *Artibeus*, *Brachyphylla*, *Carollia*, *Coleura*, *Desmodus*, *Eidolon*, *Glossophaga*. *Hipposideros*, *Lonchophylla*, *Micronycteris*, *Mimon*, *Miniopterus*, *Monophyllus*, *Myotis*, *Nyctalus*, *Otomops*, *Phyllostomus*, *Promops*, *Pteronotus*, *Rousettus*, *Rhinophylla*, *Sturnira*, *Triaenops*, *Uroderma*, and *Vampyressa* genera (Table S1, S3). Rodent hosts include individuals in the *Acomys*, *Aethomys*, *Apodemus*, *Callosciurus*, *Clethrionomys*, *Dryomys*, *Gerbillus*, *Glaucomys*, *Jaculus*, *Mastomys*, *Microtus*, *Mus*, *Myodes*, *Niviventer*, *Pachyuromys*, *Peromyscus*, *Psammomys*, *Rattus*, *Rhabdomys*, *Sekeetamys*, *Spermophilus*, *Tamias*, *Tamiasciurus*, *Tatera*, and *Urocitellus* genera (Table S2). Only unique genotypes were included in the dataset. The largest comparable genetic datasets consisted of the partial citrate synthase gene (gltA) for *Bartonella* and 16S rRNA gene for *Leptospira*, and these were selected for analysis. Cytochrome b gene sequences from all bat and rodent host species were obtained from GenBank (Tables S4, S5), as this mitochondrial gene has proven to be useful for within Order, species-level resolution of mammalian phylogenies [57]–[59]. For host species that did not have an available cytochrome b sequence, the most closely related species with available sequence was used as a substitute for host-parasite associations. For bats, we made four substitutions: *Hipposideros armiger* for *Hipposideros commersoni*, *Phyllostomus hastatus* for *Phyllostomus discolor*, *Promops centralis* for *Promops nasutus*, and *Triaenops persicus* for *Triaenops menamena*. Our results suggest that these genus-level host substitutions do not disrupt overall co-phylogenetic patterns. For all species, host taxonomy was synonymized according to Mammal Species of the World 3^rd^ Edition [60].

In total, we compiled sequences from 51 *Bartonella* genotypes (38 New World, 13 Old World) from 24 bat species (15 New World, 9 Old World), and 129 (20 New World, 109 Old World) *Bartonella* genotypes from 38 rodent species (4 New World, 35 Old World). We also compiled sequences from 26 *Leptospira* genotypes (19 New World, 7 Old World) from 20 bat species (14 New World, 6 Old World). Insufficient genetic data of one gene for *Leptospira* in rodents precluded their use in the analysis; therefore only *Leptospira* in bat hosts was examined.

### Phylogenetic analysis of sequence data

Bacterial and host species sequences were imported from GenBank into Geneious Pro 5.0.4. Sequences for each bacterial genus and their corresponding bat and rodent hosts were each aligned using default parameters in MUSCLE [61] as implemented in Geneious [62]. Outgroup taxa, obtained from GenBank, were included in each alignment, and were chosen based on previous species-level phylogenies. The outgroup for *Bartonella* was *Brucella melitensis* [25], for *Leptospira* was *Leptonema illini* [44], and for the bat and rodent hosts was the duck-billed platypus, *Ornithorhynchus anatinus* HQ379861 [63]. In order analyze the difference in host-specificity between Old and New World geographic regions, each alignment was further divided into Old and New World. Alignments were inspected visually and ends were trimmed and gaps found in only one non-outgroup sequence were deleted due to high likelihood of sequencing error. After these edits, this resulted in 1,133 base pairs (bp) for cytb bat sequences, 338 bp for gltA *Bartonella* sequences, and 1,246 bp for 16S *Leptospira* sequences.

Maximum likelihood (ML) phylogenetic trees were generated using RAxML 7.0.4 [64] implemented with the Cyberinfrastructure for Phylogenetic Research (CIPRES) Portal (www.phylo.org) using the substitution model GTRMIX, which determines an optimal tree by comparing likelihood scores under a GTR+G model. The number of bootstrap replicates were determined using the previously described stopping criteria. In order to corroborate the phylogenies as determined through ML, Bayesian inference (BI) host phylogenies were also generated using MrBayes 3.1.2 [65]. We utilized a GTR+I+G substitution model, with 10,000,000 generations, sampling every 5000^th^ generation with 4 heated chains and a burn in length of 1,000,000.

### Comparison of host and bacterial phylogenies

To visualize host-bacteria associations, tanglegrams were generated from the best ML trees in TreeMap 3.0 [66]. For cophylogenetic analyses, we utilized both global fit as well as event-based methods. We selected programs that are capable of accounting for evolutionary patterns given association of parasite species to multiple hosts, as well as the presence of multiple parasites in a single host.

Global-fit methods were used to quantify the degree of congruence between two given host and parasite topologies, and identify the individual associations contributing to the cophylogenetic structure [67]. First, global-fit analysis was tested using distance-based ParaFit [68], using matrices of patristic distances calculated from maximum likelihood host and parasite phylogenies in R 3.0.1 [69]. With an additional matrix of host-parasite links, ParaFit analyses [68] were also performed in R using package ape [70] with 999 permutations to implement a global test as well as individual links. Each individual host-bacteria interaction is determined to be significant if either its ParaFit 1 or Parafit 2 p-value≤0.05, and these significant interactions are shown in solid lines in the tanglegrams.

As ParaFit tends to be liberal with its values, we also implemented newly developed program Procrustean Approach to Cophylogeny (PACo) [71] in R using packages ape and vegan [72] in order to obtain, and potentially corroborate, comparable global goodness-of-fit statistics with Parafit global values. PACo differs from ParaFit by utilizing Procrustean superimposition, in which the parasite matrix is rotated and scaled to fit the host matrix. Thus, PACo explicitly tests the dependence of the parasite phylogeny upon the host phylogeny.

We then used event-based program Jane 4 [73] to determine the most probable coevolutionary history of the associated host and parasites, again using the ML host and bacteria trees as input. We assigned different relative costs to 5 possible evolutionary events, in a method similar to previous research efforts [74]. We performed analyses with 100 generations, population sizes of 100, and a default cost setting matrix of 0 for cospeciation, 1 for duplication of parasites, 2 for duplication and host switch, 1 for loss of parasite, and 1 for failure to diverge. In further runs, we changed one of the possible events to a cost of 10 each time, rendering that event prohibitively expensive. By further exploring the parameter space this way, we determined how these changes affected the overall costs of the optimal evolutionary history.

## Results

### Phylogenetic analysis

**Figure 1:**
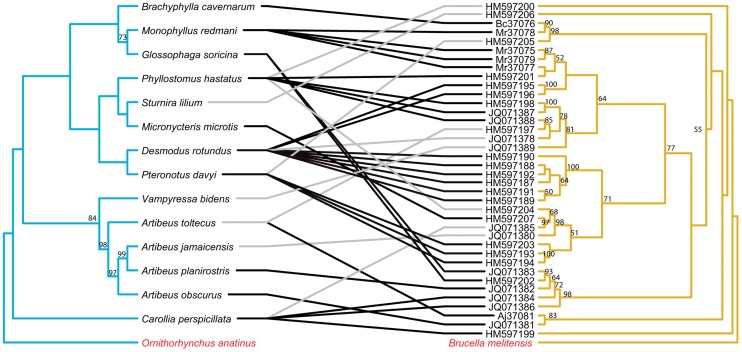
Tanglegram of cophylogenetic relationships between New World bat hosts and *Bartonella*. Maximum likelihood phylogenies for *Bartonella* bacteria (yellow) and their New World bat hosts (blue), with bootstrap support values ≥50 labeled, rooted with outgroups. All host-pathogen associations are shown in the tanglegram as gray and black connecting lines. Black lines indicate significant individual cospeciation links between *Bartonella* and their hosts as indicated by ParaFit (P≤0.05), while gray lines represent non-significant links. Bat species that did not have an available cytochrome b sequence on GenBank, is substituted with a closely related species. *Phyllostomus hastatus* substituted for *Phyllostomus discolor*.

**Figure 2:**
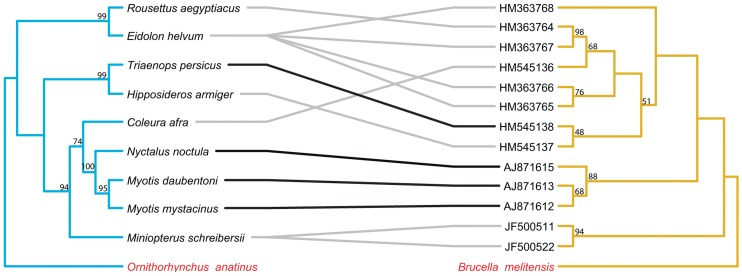
Tanglegram of cophylogenetic relationships between Old World bat hosts and *Bartonella*. Maximum likelihood phylogenies for *Bartonella* bacteria (yellow) and their Old World bat hosts (blue), with bootstrap support values ≥50 labeled, rooted with outgroups. All host-pathogen associations are shown in the tanglegram as gray and black connecting lines. Black lines indicate significant individual cospeciation links between *Bartonella* and their hosts as indicated by ParaFit (P≤0.05), while gray lines are non-significant links. Bat species that did not have an available cytochrome b sequence on GenBank, is substituted with a closely related species. *Triaenops persicus* substituted for *Triaenops menamena*, and *Hipposideros armiger* for *Hipposideros commersoni*.

**Figure 3:**
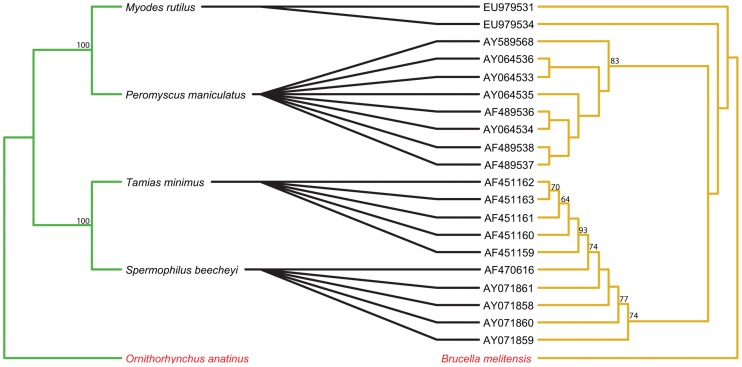
Tanglegram of cophylogenetic relationships between New World rodent hosts and *Bartonella*. Maximum likelihood phylogenies for *Bartonella* bacteria (yellow) and their New World rodent hosts (green), with bootstrap support values ≥50 labeled, rooted with outgroups. All host-pathogen associations are shown in the tanglegram as black connecting lines and are significant as indicated by ParaFit (P≤0.05).

**Figure 4:**
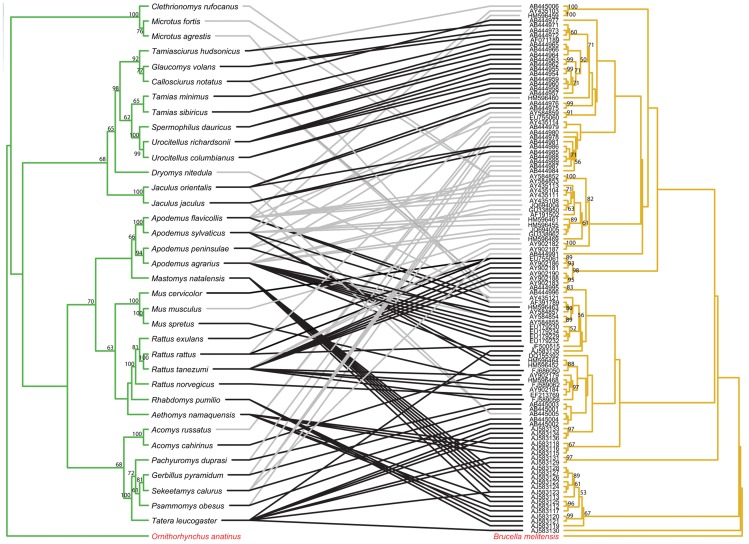
Tanglegram of cophylogenetic relationships between Old World rodent hosts and *Bartonella*. Maximum likelihood phylogenies for *Bartonella* bacteria (yellow) and their Old World rodent hosts (green), with bootstrap support values ≥50 labeled, rooted with outgroups. All host-pathogen associations are shown in the tanglegram as gray and black connecting lines. Black lines indicate significant individual cospeciation links between *Bartonella* and their hosts as indicated by ParaFit (P≤0.05), while gray lines are non-significant links.

**Figure 5:**
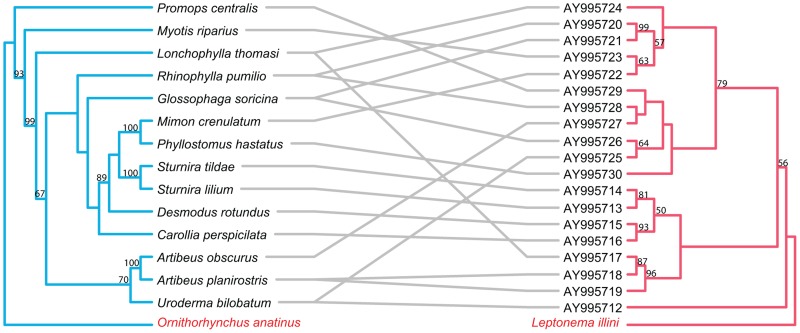
Tanglegram of cophylogenetic relationships between New World bat hosts and *Leptospira*. Maximum likelihood phylogenies for *Leptospira* bacteria (pink) and their New World bat hosts (blue), with bootstrap support values ≥50 labeled, rooted with outgroups. All host-pathogen associations are shown in the tanglegram as gray connecting lines, and are insignificant as indicated by ParaFit (P≤0.05). Bat species that did not have an available cytochrome b sequence on GenBank, is substituted with a closely related species. *Phyllostomus hastatus* substituted for *Phyllostomus discolor*, *Promops centralis* for *Promops nasutus*.

**Figure 6:**
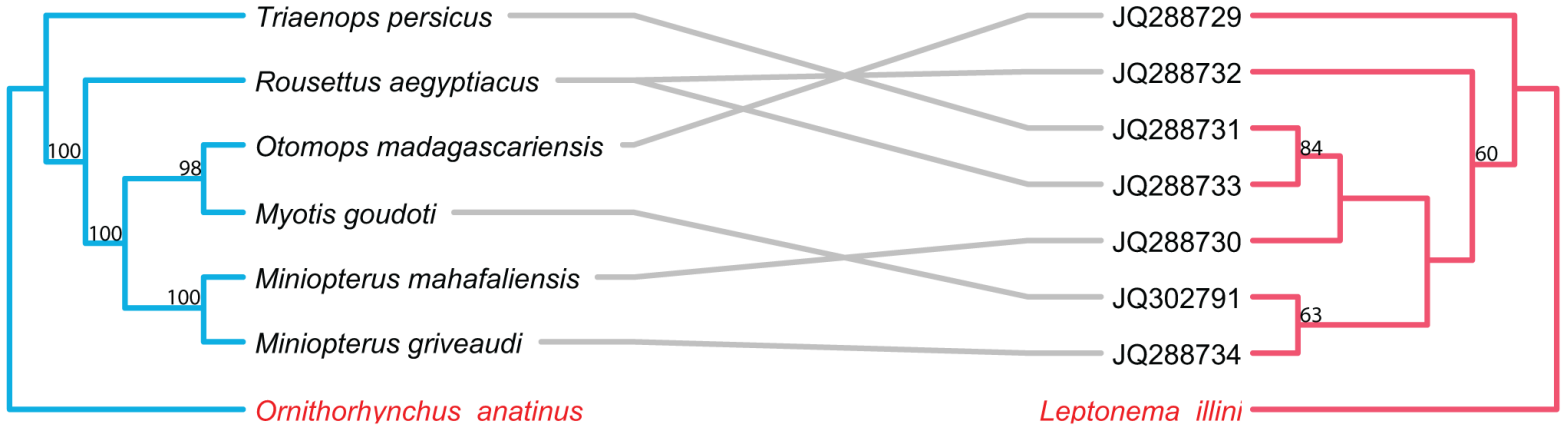
Tanglegram of cophylogenetic relationships between Old World bat hosts and *Leptospira*. Maximum likelihood phylogenies for *Leptospira* bacteria (pink) and their Old World bat hosts (blue), with bootstrap support values ≥50 labeled, rooted with outgroups. All host-pathogen associations are shown in the tanglegram as gray connecting lines, and are significant as indicated by ParaFit (P≤0.05). Bat species that did not have an available cytochrome b sequence on GenBank, is substituted with a closely related species. *Triaenops persicus* substituted for *Triaenops menamena*.

The topology of the BI tree was identical to that of the ML tree, except for a few branches with low support values. Thus, only the ML trees are presented here and used for further cophylogenetic analyses. Phylogenies tend to be well supported for more recent divergence events, but not deeper nodes. Nodes with bootstrap values ≥50 are labeled on all tanglegrams (Figures 1–6).

### Global-fit cophylogeny

#### Bats–*Bartonella*

Overall, both ParaFit and PACo analyses of *Bartonella* and bats provided evidence for significant co-evolution between *Bartonella* and bat hosts (ParaFitGlobal = 1.9703, P≤0.001; m^2^ global value = 7.3320, P≤0.0001). Twenty-six of the 51 individual host-parasite links are significant based on either a ParaFit1 or Parafit2 value of P≤0.05. While there was evidence of overall cospeciation, a substantial proportion of specific host-parasite links were non-significant.

The separate analyses of New World and Old World bat associated *Bartonella* both indicated evidence for overall coevolution with host species. In the New World dataset, global values from both ParaFit and PACo were significant (ParaFitGlobal = 0.4762, P≤0.001; m^2^ global value = 2.3313, P≤0.0001) and 29/38 individual were significant (Figure 1). For the Old World dataset, (ParaFitGlobal = 0.0871, P = 0.029; m^2^ global value = 0.7385, P = 0.0004) and 4/13 individual links were significant (Figure 2).

#### Rodents–*Bartonella*

Both ParaFit and PACo analyses of *Bartonella* and rodent phylogenies also indicated strong overall patterns of cospeciation (ParaFitGlobal = 102.4409, P≤0.001; m^2^ global value = 86.532, P≤0.0001). For the entire rodent-*Bartonella* dataset, 94/140 individual host-parasite links were significant based on ParaFit1 values and P≤0.05.

The separate analyses for the New World and Old World rodent hosts with associated *Bartonella* both also indicated evidence for significant overall coevolution. In the New World dataset, global values from both ParaFit and PACo were significant (ParaFitGlobal = 0.156, P≤0.001; m^2^ global value = 0.3548, P≤0.0001) and all 20 individual links were significant (Figure 3). For the Old World dataset, (ParaFitGlobal = 42.8037, P≤0.001; m^2^ global value = 72.31235, P≤0.0001) and 79/120 individual links were significant (Figure 4).

#### Bats–*Leptospira*

In contrast to *Bartonella* in mammalian hosts, ParaFit and PACo analyses of *Leptospira* and bats were unable to reject the hypothesis of independence of speciation events (ParaFitGlobal = 0.0042, P = 0.84; m^2^ global value = 4.6310, P = 0.5629). Based on ParaFit1 values, only 1 of 26 individual host-parasite links is significant based on P≤0.05. The separate analyses for the New World and Old World bat hosts with associated *Bartonella* both also indicated no evidence for overall coevolution. In the New World dataset, global values from both ParaFit and PACo were non-significant (ParaFitGlobal = 0.0012, P = 0.858; m^2^ global value = 4.631, P = 0.563) and none of the 19 individual links were significant (Figure 5). For the Old World dataset, (ParaFitGlobal = 0.0002, P = 0.269; m^2^ global value = 2.0802, P = 0.7587) and none of the 7 individual links were significant (Figure 6).

### Event-based cophylogeny

Based on a default cost setting, we calculated the optimal number of each type of coevolutionary event, to minimize total cost, for each host-pathogen association (Table 1). In order to account for the different sample sizes of each of the phylogenies, we divided the number of cospeciation and host switch events by the number of parasites in each association (Table 1). The resulting ratios can then be compared across the different associations in order to see the overall impact of each event given the number of parasites. Based on these calculations, *Leptospira* and bat host associations have the greatest number of host switches per parasite (0.731), while *Bartonella* and rodent host associations have the fewest (0.264). *Leptospira* and bat host associations also have the greatest number of cospeciations per parasite (0.231), while *Bartonella* and rodent host associations have the fewest (0.132).

**Table 1:** Results from event-based cophylogeny, using default cost settings of 0, 1, 2, 1, 1 in Jane.

	Cospeciations	Duplications	Duplications and host switches	Losses	Failures to diverge	Total Cost	# of hosts	# of parasites	Host switches/parasites	Cospeciations/parasites
***Bats-Bartonella***	10	21	24	4	0	73	23	56	0.429	0.179
*New World*	5	14	18	3	0	53	14	38	0.474	0.132
*Old World*	6	6	5	1	0	17	9	18	0.278	0.333
***Bats-Leptospira***	6	0	19	3	0	41	20	26	0.731	0.231
*New World*	5	1	12	2	0	27	14	19	0.632	0.263
*Old World*	2	0	4	1	0	9	6	7	0.571	0.286
***Rodents-Bartonella***	16	77	35	127	11	285	39	129	0.264	0.132
*New World*	1	15	3	0	0	21	4	20	0.150	0.050
*Old World*	17	54	37	75	11	214	35	109	0.339	0.156

We also compared cophylogenetic fit between bacteria and Old World vs. New World host species. *Bartonella* had nearly twice as many host switches per parasite in New World (0.474) as compared to Old World bats (0.278). *Bartonella* in rodents had the opposite trend, with more than twice as many host switches per parasite in Old World (0.339) as compared to New World rodents (0.150). There were also approximately three times as many cospeciation events per parasite for *Bartonella* in the Old World for both groups of hosts (bats: 0.333, rodents: 0.156) compared to the New World (bats: 0.132, rodents: 0.050). For *Leptospira*, the differences between Old and New World cophylogenetic patterns for both host switches and cospeciations were minimal.

We explored a wide-range of cost parameters in order to determine the effect of removing different evolutionary event options from each analysis (Table S6). Increasing the cost of host switching events had the greatest overall impact on total cost. In fact, the role of the host switching events in the overall coevolutionary pattern was so strong that even with a potentially prohibitive cost of 10, the solution still proposed between 2–4 host switch events for *Bartonella* in New World bats and Old World rodents.

## Discussion

We found significant coevolutionary congruence between *Bartonella* and both their rodent and bat hosts at a global level, while the relationship between *Leptospira* and their bat hosts was non-significant. Event-cost results support the global-fit findings, with the rodent-*Bartonella* and bat-*Bartonella* associations having the least number of host switches per parasite, which indicates greater evolutionary congruence over time. Co-evolution of bartonellae and their mammalian hosts also remains significant when New and Old World datasets are analyzed separately. The evolutionary pattern in bat hosts is driven mostly by a few strong host-parasite interactions, with 51% of individual associations significant. In comparison, a greater proportion, 67%, of the individual rodent-bacteria associations are significant, indicating stronger coevolutionary interactions throughout these lineages. In fact, in the New World association of rodents and *Bartonella*, a full 100% of the host-parasite links were significant. In contrast, for *Leptospira* and their bat hosts, there is only 1 significant individual in analysis of the entire data set, and no significant host-parasite links when the data are analyzed separately as Old vs. New World. We note that the sample sizes for *Bartonella* in New World rodents and *Leptospira* in Old World bats are both small, and that the observed patterns could change with the addition of more data. Similarly, the relatively short sequence available of only one gene for both *Bartonella* and *Leptospira* may limit the resolution and nodal support for the pathogen phylogenies we obtained. These issues can only be addressed with additional sampling and genetic sequencing to complement these sparse datasets. For example, while our analysis of *Bartonella*-host relationships was limited by the availability of glt*A* fragments, the use of multi-gene phylogenies would be a more robust approach given the confounding effect of recombination [75]. Despite these potentially confounding factors, our preliminary analysis suggests a strong signal was present for some host-pathogen relationships and at a host order and pathogen genus level these trends were generalizable.

Event-cost methods corroborate the non-significant coevolutionary history of *Leptospira* and bats. Interestingly, the number of cospeciations per parasite is also the highest for *Leptospira* and bats, although they also have the highest number of host switches per parasite. Since their overall coevolutionary relationship is nonsignificant, this suggests that for the bat-*Leptospira* system, coevolutionary relationships are driven mostly strongly by the host switching events rather than cospeciation. Exploring the parameter space of cost structures further supports our findings. For all associations, maximizing the cost of host switching results in the largest overall change in the total cost (Table S6). This indicates that host switching is an “expensive” evolutionary event, and our finding of frequent and well-supported host switching in the bat-*Leptospira* system suggest that there are intrinsic ecological and transmission factors driving this.

One explanation for the different coevolutionary patterns between *Bartonella* and *Leptopira* may be differences in the modes of transmission and infection dynamics for each pathogen. As a vector transmitted parasite, *Bartonella* has an additional evolutionary step in adapting to an arthropod organism as well as a mammalian host. Combined, this can exert greater evolutionary selection and act as a selective force driving speciation. Further, *Bartonella* forms persistent, often asymptomatic, infections in its hosts [17], and some evidence even suggests that *Bartonella* may be acting as a symbiont more than a pathogen [18], [76], [77]. Many *Bartonella* species are also likely transmitted by only one arthropod species [78], and this specificity can then be translated to a greater coevolutionary pattern between the disease and eventual mammalian host. In bats, the arthropod vectors include blood-feeding bat flies, from which *Bartonella* has been sequenced and cultured [77], [79]. Host specificity of these arthropods may help to maintain the high diversity of *Bartonella* and long-term coevolutionary patterns between bat flies and their *Bartonella* parasites [77]. However no in-depth cophylogenetic analyses have been conducted for these bacteria and their known arthropod vectors, and this is an area for future exploration. Additional studies on arthropod ecology, e.g. bat fly, population structure, dispersal, ecology, and host specificity will also help to clarify the role of bat hosts vs. arthropod vectors in the evolution of *Bartonella* [77], [80]. Additionally, Bartonella is an intracellular bacteria which can survive only within erythrocytes and endothelial cells [17]. This requires a finer adaptation to the host's cells in order for bacterial penetration. In summary, *Bartonella* infection dynamics favor vector transmission, and the specific host-vector relationships, potential vertical transmission in vectors, and intracellular nature of the bacteria allow for co-evolutionary relationships to develop over time.

In contrast, *Leptospira* spp. are not vector-transmitted and instead are transmitted via environmental contamination. Leptospires are able to survive outside of their hosts, and can persist in water bodies when shed in animal urine [81]. Although the vast majority of *Leptospira* infections are mild, a small proportion involve multiple organ systems and develop various complications resulting in a case fatality in human patients of about 40% [45]. As contact with urine and contaminated water is the main form of disease transmission, physical proximity to environmental sources can play a large role in influencing host-pathogen interactions [82]. Thus it is possible that geographic overlap of the host species will better predict similarity in the bacteria they carry rather than the phylogenetic relatedness of the hosts. Overlapping geographic distribution of host species has been found to be an important determinant of pathogen sharing in primates [83]. The role of environmental transmission is most likely why we observed frequent host switching events and a lack of coevolutionary patterns in the *Leptospira* lineages we studied. Further investigations of Leptospirosis disease dynamics, including shedding, transmission, and immunity, in bat populations is warranted, as well as their zoonotic potential given the propensity towards cross-species transmission.

We originally hypothesized a difference in the strength of coevolutionary relationships between Old and New World host species, since previous research in bats had indicated host specificity in the Old World but not New World for *Bartonella* [24]. While the mechanism for this observation was not clear, it may be hypothesized that a greater degree of congruence between host-bacteria phylogenies in the Old World may be due to longer evolutionary time for the establishment of mutualistic relationships with mammalian hosts [24]. Yet, in our larger datasets, we did not see this pattern emerge, and our results indicated that coevolutionary patterns are generalizable globally. For *Bartonella*, significant coevolutionary congruence with hosts was evident globally and across host ranges, while for *Leptospira*, the lack of a coevolutionary relationship in bat hosts was evident in both the Old and New World. However, it is interesting that we observed a stronger relationship between rodents and *Bartonella* than between bats and *Bartonella*. There are two possible explanations for this. First, in mammalian evolutionary history, rodents existed for a longer period with 4.1 million years earlier time of origin and a 10 million year difference in time of basal diversification between the two [9]. Thus it is possible that there has been a longer time for parasite-host relations to coevolve in rodents and create stronger patterns. However it is not clear that these hosts have been infected with the two pathogens in question over their entire evolutionary history, and further detecting such deep evolutionary divergences is confounded by genetic saturation and nucleotide homoplasy. A second explanation is that the ecological differences between bats and rodents may explain the observed differences in host-specificity. Unlike rodents, a number of bat species form large multi-species gatherings and are more likely to have direct ecological overlap between host species (e.g. many thousands of individuals from >8 bat species roosting together in a single cave site in Mexico [84]). Similarly, at sites across the tropics, an extraordinary numbers of bat species can exist in sympatry, e.g. >70 species sharing tropical forest habitat in Krau Wildlife Reserve, Malaysia [85]. The gregarious aggregations of highly mobile individuals, often between multiple species, may help to explain differences in the global coevolutionary patterns observed between bats and rodents. While there has been growing scientific interest in these ecological and life-history host traits to explain viral sharing in bats and rodents [5], [8], [10], the role that these traits may play in bacterial pathogen diversification and spillover has been little investigated to date.

Overall, it is likely that the interplay of multiple factors, including geographic overlap, pathogen transmission pathways, infection dynamics, and host ecological and evolutionary history, that contribute to the contrasting coevolutionary patterns evident in mammal-bacteria interactions we observed. Further research is warranted to better understand and tease apart these contributing factors, and we recognize some of the limitations of this preliminary study. First, our analysis was limited by the availability of comparable data sets for a given gene and host taxonomic group. This precluded us from examining *Leptospira* in rodents; and resulted in low support values from some nodes in our phylogenies. In the future, using multiple genes or full genome data, for a greater number of bat and rodent taxonomic groups and bacterial microbes once they are available, will allow for more robust taxonomic analyses. Also, in addition to host phylogeny that we examine here, future data collection and analyses should focus on arthropod vector host specificity and phylogenetic relationships to better predict specificity within *Bartonella*. Future investigations should also consider the role of host geographic range and niche overlap to explain pathogen sharing between hosts. The application of spatial analyses of wildlife hosts for both *Bartonella* and *Leptospira* will provide valuable information on transmission potential based on the role of contact vs. cophylogeny. We predict that species with overlapping ranges will share more similar communities of *Leptospira* than non-overlapping bat species, regardless of their phylogenetic relatedness. For *Bartonella* there is also likely to be a geographic effect, as interaction among bats of different species within multi-species roosts, or shared habitats, could be an important factor for bacterial pathogen sharing.

Lastly, this work is particularly important because it involves two emerging, neglected tropical diseases with known, sylvatic wildlife reservoirs. *Bartonella* has been of concern as an emerging zoonoses due to its ability to induce life-threatening illnesses such as endocarditis, myocarditis, meningoencephalitis, and contributing to chronic debilitating disease, all while being difficult to diagnose in humans as well as animals [86]. Leptospirosis is a constant concern to public health authorities, and annual global incidence of severe leptospirosis has been estimated as 500,000 [87]. Elucidating the diversity and coevolutionary patterns of these bacteria in their natural hosts and understanding the frequency and causes of host-switching events, will help us better predict spillover from the mammal reservoirs into humans. Disruption of strict coevolutionary patterns, as we observed for both bacterial genera, to varying degrees, provides a framework to forecast pathogen spillover potential to any mammalian host, including humans [88]. The methods that we employed here to study bacterial disease in bats and rodent hosts are broadly applicable to a wide range of other disease types, including viruses in their mammalian hosts. By expanding these tools to better understand the evolutionary past of pathogens within and among wildlife hosts, we gain information to better predict the outbreaks of the future.

## Supporting Information

Figure S1 **Disease-related publications for bats and rodents over the past 20 years.** The number of publications pertaining to viral (red) and bacterial (blue) disease research in bats (dotted) and rodents (solid) in each year from 1993 to 2012. Data from Web of Science keyword search for all publications from 1993 onwards using combinations of keywords bat OR Chiroptera*, rodent*, bacteria*, virus* OR vira*. Bacterial studies in bats are the most understudied category to date.
(DOCX)

Table S1 **gltA GenBank accession numbers for studied** ***Bartonella*** **sequences in bat hosts.**
(DOCX)

Table S2 **gltA GenBank accession numbers for studied** ***Bartonella*** **sequences in rodent hosts.**
(DOCX)

Table S3 **16S GenBank accession numbers for studied** ***Leptospira*** **sequences in bat hosts.**
(DOCX)

Table S4 **Cytochrome b GenBank accession numbers of bat species host to studied bacteria.**
(DOCX)

Table S5 **Cytochrome b GenBank accession numbers of rodent species host to studied** ***Bartonella.***
(DOCX)

Table S6 **Results from event-based cophylogeny Jane, using different cost-settings.**
(DOCX)
