# Supplementary material for: Contrasting Patterns in Mammal–Bacteria Coevolution: Bartonella and Leptospira in Bats and Rodents
Source: PLoS Negl Trop Dis. 2014 Mar 20;8(3):e2738. doi: 10.1371/journal.pntd.0002738 (PMC3961187; doi:10.1371/journal.pntd.0002738)
Supplement: Figure S1 — Disease-related publications for bats and rodents over the past 20 years. The number of publications pertaining to viral (red) and bacterial (blue) disease research in bats (dotted) and rodents (solid) in each year from 1993 to 2012. Data from Web of Science keyword search for all publications from 1993 onwards using combinations of keywords bat OR Chiroptera*, rodent*, bacteria*, virus* OR vira*. Bacterial studies in bats are the most understudied category to date. (DOCX) [file pntd.0002738.s001.docx]

**
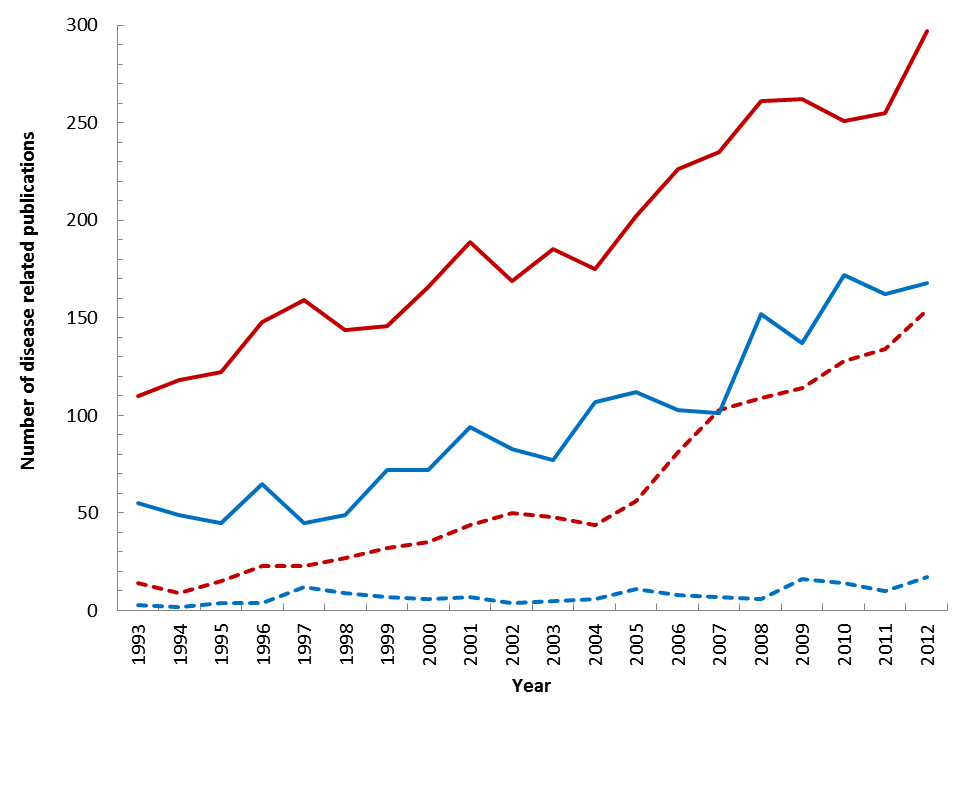
**

**Figure S1.** **Disease-related publications for bats and rodents over the past 20 years**

The number of publications pertaining to viral (red) and bacterial (blue) disease research in bats (dotted) and rodents (solid) in each year from 1993 to 2012. Data from Web of Science keyword search for all publications from 1993 onwards using combinations of keywords bat OR Chiroptera*, rodent*, bacteria*, virus* OR vira*. Bacterial studies in bats are the most understudied category to date.
