# Supplementary material for: Contrasting Patterns in Mammal–Bacteria Coevolution: Bartonella and Leptospira in Bats and Rodents
Source: PLoS Negl Trop Dis. 2014 Mar 20;8(3):e2738. doi: 10.1371/journal.pntd.0002738 (PMC3961187; doi:10.1371/journal.pntd.0002738)
Supplement: Table S1 — gltA GenBank accession numbers for studied Bartonella sequences in bat hosts. (DOCX) [file pntd.0002738.s002.docx]

**Table S1.** gltA GenBank accession numbers for studied *Bartonella* sequences in bat hosts

| GenBank Accession | Host Species | Paper | Country |
| --- | --- | --- | --- |
| AJ871612 | *Myotis mystacinus* | Concannon et al. 2005 | UK |
| AJ871613 | *Myotis daubentoni* | Concannon et al. 2005 | UK |
| AJ871615 | *Nyctalus noctula* | Concannon et al. 2005 | UK |
| HM363764 | *Rousettus aegyptiacus* | Kosoy et al. 2010 | Kenya |
| HM363765 | *Eidolon helvum* | Kosoy et al. 2010 | Kenya |
| HM363766 | *Eidolon helvum* | Kosoy et al. 2010 | Kenya |
| HM363767 | *Eidolon helvum* | Kosoy et al. 2010 | Kenya |
| HM363768 | *Eidolon helvum* | Kosoy et al. 2010 | Kenya |
| HM545136 | *Coleura afra* | Kosoy et al. 2010 | Kenya |
| HM545137 | *Hipposideros commersoni** | Kosoy et al. 2010 | Kenya |
| HM545138 | *Triaenops persicus* | Kosoy et al. 2010 | Kenya |
| HM597187 | *Desmodus rotundus* | Bai et al. 2011 | Guatemala |
| HM597188 | *Desmodus rotundus* | Bai et al. 2011 | Guatemala |
| HM597189 | *Desmodus rotundus* | Bai et al. 2011 | Guatemala |
| HM597190 | *Desmodus rotundus* | Bai et al. 2011 | Guatemala |
| HM597191 | *Desmodus rotundus* | Bai et al. 2011 | Guatemala |
| HM597192 | *Desmodus rotundus* | Bai et al. 2011 | Guatemala |
| HM597193 | *Pteronotus davyi* | Bai et al. 2011 | Guatemala |
| HM597194 | *Pteronotus davyi* | Bai et al. 2011 | Guatemala |
| HM597195 | *Desmodus rotundus* | Bai et al. 2011 | Guatemala |
| HM597196 | *Desmodus rotundus* | Bai et al. 2011 | Guatemala |
| HM597197 | *Artibeus toltecus* | Bai et al. 2011 | Guatemala |
| HM597198 | *Phyllostomus discolor** | Bai et al. 2011 | Guatemala |
| HM597199 | *Carollia perspicillata* | Bai et al. 2011 | Guatemala |
| HM597200 | *Phyllostomus discolor** | Bai et al. 2011 | Guatemala |
| HM597201 | *Phyllostomus discolor** | Bai et al. 2011 | Guatemala |
| HM597202 | *Glossophaga soricina* | Bai et al. 2011 | Guatemala |
| HM597203 | *Pteronotus davyi* | Bai et al. 2011 | Guatemala |
| HM597204 | *Phyllostomus discolor** | Bai et al. 2011 | Guatemala |
| HM597205 | *Pteronotus davyi* | Bai et al. 2011 | Guatemala |
| HM597206 | *Sturnira lilium* | Bai et al. 2011 | Guatemala |
| HM597207 | *Micronycteris microtis* | Bai et al. 2011 | Guatemala |
| JF500511 | *Miniopterus schreibersii* | Lin et al. 2012 | Taiwan |
| JF500522 | *Miniopterus schreibersii* | Lin et al. 2012 | Taiwan |
| JQ071378 | *Desmodus rotundus* | Bai et al. 2012 | Peru |
| JQ071380 | *Artibeus obscurus* | Bai et al. 2012 | Peru |
| JQ071381 | *Artibeus planirostris* | Bai et al. 2012 | Peru |
| JQ071382 | *Artibeus planirostris* | Bai et al. 2012 | Peru |
| JQ071383 | *Glossophaga soricina* | Bai et al. 2012 | Peru |
| JQ071384 | *Carollia perspicillata* | Bai et al. 2012 | Peru |
| JQ071385 | *Carollia perspicillata* | Bai et al. 2012 | Peru |
| JQ071386 | *Carollia perspicillata* | Bai et al. 2012 | Peru |
| JQ071387 | *Phyllostomus discolor** | Bai et al. 2012 | Peru |
| JQ071388 | *Phyllostomus hastatus* | Bai et al. 2012 | Peru |
| JQ071389 | *Vampyressa bidens* | Bai et al. 2012 | Peru |
| Aj37081 | *Artibeus jamaicensis* | Olival, unpublished | Puerto Rico |
| Bc37076 | *Brachyphylla cavernarum* | Olival, unpublished | Puerto Rico |
| Mr37075 | *Monophyllus redmani* | Olival, unpublished | Puerto Rico |
| Mr37077 | *Monophyllus redmani* | Olival, unpublished | Puerto Rico |
| Mr37078 | *Monophyllus redmani* | Olival, unpublished | Puerto Rico |
| Mr37079 | *Monophyllus redmani* | Olival, unpublished | Puerto Rico |
