# Supplementary material for: Contrasting Patterns in Mammal–Bacteria Coevolution: Bartonella and Leptospira in Bats and Rodents
Source: PLoS Negl Trop Dis. 2014 Mar 20;8(3):e2738. doi: 10.1371/journal.pntd.0002738 (PMC3961187; doi:10.1371/journal.pntd.0002738)
Supplement: Table S2 — gltA GenBank accession numbers for studied Bartonella sequences in rodent hosts. (DOCX) [file pntd.0002738.s003.docx]

**Table S2.** gltA GenBank accession numbers for studied *Bartonella* sequences in rodent hosts

| GenBank number | Host Species | Paper | Country |
| --- | --- | --- | --- |
| AB444954 | *Urocitellus richardsonii* | Inoue et al. 2009 | Unknown - imported into Japan |
| AB444955 | *Urocitellus richardsonii* | Inoue et al. 2009 | Unknown - imported into Japan |
| AB444957 | *Urocitellus columbianus* | Inoue et al. 2009 | Unknown - imported into Japan |
| AB444958 | *Urocitellus columbianus* | Inoue et al. 2009 | Unknown - imported into Japan |
| AB444959 | *Urocitellus richardsonii* | Inoue et al. 2009 | Unknown - imported into Japan |
| AB444960 | *Urocitellus richardsonii* | Inoue et al. 2009 | Unknown - imported into Japan |
| AB444962 | *Spermophilus dauricus* | Inoue et al. 2009 | Unknown - imported into Japan |
| AB444963 | *Spermophilus dauricus* | Inoue et al. 2009 | Unknown - imported into Japan |
| AB444964 | *Tamias sibiricus* | Inoue et al. 2009 | Unknown - imported into Japan |
| AB444965 | *Tamias sibiricus* | Inoue et al. 2009 | Unknown - imported into Japan |
| AB444966 | *Tamias sibiricus* | Inoue et al. 2009 | Unknown - imported into Japan |
| AB444971 | *Tamiasciurus hudsonicus* | Inoue et al. 2009 | Unknown - imported into Japan |
| AB444972 | *Glaucomys volans* | Inoue et al. 2009 | Unknown - imported into Japan |
| AB444973 | *Glaucomys volans* | Inoue et al. 2009 | Unknown - imported into Japan |
| AB444975 | *Jaculus orientalis* | Inoue et al. 2009 | Unknown - imported into Japan |
| AB444976 | *Jaculus orientalis* | Inoue et al. 2009 | Unknown - imported into Japan |
| AB444977 | *Callosciurus notatus* | Inoue et al. 2009 | Unknown - imported into Japan |
| AB444978 | *Pachyuromys duprasi* | Inoue et al. 2009 | Unknown - imported into Japan |
| AB444979 | *Acomys russatus* | Inoue et al. 2009 | Unknown - imported into Japan |
| AB444980 | *Acomys russatus* | Inoue et al. 2009 | Unknown - imported into Japan |
| AB444981 | *Gerbillus pyramidum* | Inoue et al. 2009 | Unknown - imported into Japan |
| AB444984 | *Psammomys obesus* | Inoue et al. 2009 | Unknown - imported into Japan |
| AB444985 | *Jaculus jaculus* | Inoue et al. 2009 | Unknown - imported into Japan |
| AB444986 | *Jaculus jaculus* | Inoue et al. 2009 | Unknown - imported into Japan |
| AB444986 | *Jaculus orientalis* | Inoue et al. 2009 | Unknown - imported into Japan |
| AB444987 | *Sekeetamys calurus* | Inoue et al. 2009 | Unknown - imported into Japan |
| AB444988 | *Sekeetamys calurus* | Inoue et al. 2009 | Unknown - imported into Japan |
| AB444989 | *Sekeetamys calurus* | Inoue et al. 2009 | Unknown - imported into Japan |
| AB444991 | *Sekeetamys calurus* | Inoue et al. 2009 | Unknown - imported into Japan |
| AB444995 | *Callosciurus notatus* | Inoue et al. 2009 | Unknown - imported into Japan |
| AB444996 | *Callosciurus notatus* | Inoue et al. 2009 | Unknown - imported into Japan |
| AB445001 | *Gerbillus pyramidum* | Inoue et al. 2009 | Unknown - imported into Japan |
| AB445002 | *Psammomys obesus* | Inoue et al. 2009 | Unknown - imported into Japan |
| AB445003 | *Pachyuromys duprasi* | Inoue et al. 2009 | Unknown - imported into Japan |
| AB445004 | *Sekeetamys calurus* | Inoue et al. 2009 | Unknown - imported into Japan |
| AB445005 | *Jaculus jaculus* | Inoue et al. 2009 | Unknown - imported into Japan |
| AB445005 | *Jaculus orientalis* | Inoue et al. 2009 | Unknown - imported into Japan |
| AB445006 | *Tamiasciurus hudsonicus* | Inoue et al. 2009 | Unknown - imported into Japan |
| AF071189 | *Tamias minimus* | Regnery et al. 1998 | Greece |
| AF191502 | *Apodemus sylvaticus* | Tea et al. 2004 | Greece |
| AF391789 | *Apodemus flavicollis* | Tea et al. 2004 | Greece |
| AF391789 | *Microtus agrestis* | Tea et al. 2004 | Greece |
| AF451159 | *Tamias minimus* | Kosoy et al. 2003 | United States |
| AF451160 | *Tamias minimus* | Kosoy et al. 2003 | United States |
| AF451161 | *Tamias minimus* | Kosoy et al. 2003 | United States |
| AF451162 | *Tamias minimus* | Kosoy et al. 2003 | United States |
| AF451163 | *Tamias minimus* | Kosoy et al. 2003 | United States |
| AF470616 | *Spermophilus beecheyi* | Kosoy et al. 2003 | United States |
| AF489536 | *Peromyscus maniculatus* | Kosoy et al. 2003 | United States |
| AF489537 | *Peromyscus maniculatus* | Kosoy et al. 2003 | United States |
| AF489538 | *Peromyscus maniculatus* | Kosoy et al. 2003 | United States |
| AJ583112 | *Aethomys namaquensis* | Pretorius et al. 2004 | South Africa |
| AJ583113 | *Aethomys namaquensis* | Pretorius et al. 2004 | South Africa |
| AJ583115 | *Aethomys namaquensis* | Pretorius et al. 2004 | South Africa |
| AJ583116 | *Aethomys namaquensis* | Pretorius et al. 2004 | South Africa |
| AJ583117 | *Aethomys namaquensis* | Pretorius et al. 2004 | South Africa |
| AJ583118 | *Aethomys namaquensis* | Pretorius et al. 2004 | South Africa |
| AJ583119 | *Rhabdomys pumilio* | Pretorius et al. 2004 | South Africa |
| AJ583120 | *Rhabdomys pumilio* | Pretorius et al. 2004 | South Africa |
| AJ583121 | *Rhabdomys pumilio* | Pretorius et al. 2004 | South Africa |
| AJ583122 | *Mastomys natalensis* | Pretorius et al. 2004 | South Africa |
| AJ583123 | *Mastomys natalensis* | Pretorius et al. 2004 | South Africa |
| AJ583123 | *Tatera leucogaster* | Pretorius et al. 2004 | South Africa |
| AJ583124 | *Mastomys natalensis* | Pretorius et al. 2004 | South Africa |
| AJ583125 | *Mastomys natalensis* | Pretorius et al. 2004 | South Africa |
| AJ583126 | *Mastomys natalensis* | Pretorius et al. 2004 | South Africa |
| AJ583127 | *Mastomys natalensis* | Pretorius et al. 2004 | South Africa |
| AJ583128 | *Mastomys natalensis* | Pretorius et al. 2004 | South Africa |
| AJ583129 | *Tatera leucogaster* | Pretorius et al. 2004 | South Africa |
| AJ583130 | *Tatera leucogaster* | Pretorius et al. 2004 | South Africa |
| AJ583131 | *Tatera leucogaster* | Pretorius et al. 2004 | South Africa |
| AJ583133 | *Tatera leucogaster* | Pretorius et al. 2004 | South Africa |
| AJ583134 | *Tatera leucogaster* | Pretorius et al. 2004 | South Africa |
| AJ583135 | *Tatera leucogaster* | Pretorius et al. 2004 | South Africa |
| AJ583136 | *Tatera leucogaster* | Pretorius et al. 2004 | South Africa |
| AY064533 | *Peromyscus maniculatus* | Kosoy et al. 2003 | United States |
| AY064534 | *Peromyscus maniculatus* | Kosoy et al. 2003 | United States |
| AY064535 | *Peromyscus maniculatus* | Kosoy et al. 2003 | United States |
| AY064536 | *Peromyscus maniculatus* | Kosoy et al. 2003 | United States |
| AY071858 | *Spermophilus beecheyi* | Kosoy et al. 2003 | United States |
| AY071859 | *Spermophilus beecheyi* | Kosoy et al. 2003 | United States |
| AY071860 | *Spermophilus beecheyi* | Kosoy et al. 2003 | United States |
| AY071861 | *Spermophilus beecheyi* | Kosoy et al. 2003 | United States |
| AY435103 | *Apodemus flavicollis* | Tea et al. 2004 | Greece |
| AY435104 | *Apodemus flavicollis* | Tea et al. 2004 | Greece |
| AY435108 | *Apodemus flavicollis* | Tea et al. 2004 | Greece |
| AY435111 | *Apodemus flavicollis* | Tea et al. 2004 | Greece |
| AY435113 | *Apodemus flavicollis* | Tea et al. 2004 | Greece |
| AY435114 | *Apodemus flavicollis* | Tea et al. 2004 | Greece |
| AY435121 | *Apodemus flavicollis* | Tea et al. 2004 | Greece |
| AY435121 | *Dryomys nitedula* | Tea et al. 2004 | Greece |
| AY584852 | *Apodemus peninsulae* | Mediannikov et al. 2005 | Russia |
| AY584852 | *Clethrionomys rufocanus* | Mediannikov et al. 2005 | Russia |
| AY584852 | *Microtus fortis* | Mediannikov et al. 2005 | Russia |
| AY584853 | *Apodemus peninsulae* | Mediannikov et al. 2005 | Russia |
| AY584854 | *Apodemus agrarius* | Mediannikov et al. 2005 | Russia |
| AY584854 | *Apodemus peninsulae* | Mediannikov et al. 2005 | Russia |
| AY584855 | *Apodemus agrarius* | Mediannikov et al. 2005 | Russia |
| AY584857 | *Apodemus agrarius* | Mediannikov et al. 2005 | Russia |
| AY584859 | *Apodemus agrarius* | Mediannikov et al. 2005 | Russia |
| AY584859 | *Apodemus peninsulae* | Mediannikov et al. 2005 | Russia |
| AY589568 | *Peromyscus maniculatus* | Bai et al. 2011 | United States |
| AY902179 | *Rattus tanezumi* | Winoto et al. 2005 | Indonesia |
| AY902181 | *Rattus tanezumi* | Winoto et al. 2005 | Indonesia |
| AY902182 | *Rattus tanezumi* | Winoto et al. 2005 | Indonesia |
| AY902183 | *Rattus tanezumi* | Winoto et al. 2005 | Indonesia |
| AY902184 | *Rattus tanezumi* | Winoto et al. 2005 | Indonesia |
| AY902186 | *Rattus tanezumi* | Winoto et al. 2005 | Indonesia |
| AY902187 | *Rattus tanezumi* | Winoto et al. 2005 | Indonesia |
| AY902188 | *Rattus tanezumi* | Winoto et al. 2005 | Indonesia |
| AY902190 | *Rattus tanezumi* | Winoto et al. 2005 | Indonesia |
| DQ155392 | *Apodemus agrarius* | Hildebrand et al. 2013 | Poland |
| EF213769 | *Rattus norvegicus* | unpublished | China |
| EU179229 | *Apodemus agrarius* | Liu et al. 2010 | China |
| EU179230 | *Apodemus agrarius* | Liu et al. 2010 | China |
| EU179232 | *Apodemus agrarius* | Liu et al. 2010 | China |
| EU179234 | *Apodemus agrarius* | Liu et al. 2010 | China |
| EU755060 | *Rattus rattus* | Angelakis et al. 2009 | Laos |
| EU755061 | *Mus cervicolor* | Angelakis et al. 2009 | Laos |
| EU755061 | *Rattus exulans* | Angelakis et al. 2009 | Laos |
| EU755061 | *Rattus rattus* | Angelakis et al. 2009 | Laos |
| EU979531 | *Myodes rutilus* | Matsumoto et al. 2010 | United States |
| EU979534 | *Myodes rutilus* | Matsumoto et al. 2010 | United States |
| FJ589056 | *Rattus tanezumi* | unpublished | China |
| FJ589062 | *Rattus tanezumi* | unpublished | China |
| FJ686050 | *Acomys cahirinus* | unpublished | Israel |
| GU338950 | *Apodemus agrarius* | Hildebrand et al. 2013 | Poland |
| GU338962 | *Apodemus agrarius* | Hildebrand et al. 2013 | Poland |
| HM596452 | *Apodemus sylvaticus* | Gil et al. 2010 | Spain |
| HM596455 | *Apodemus sylvaticus* | Gil et al. 2010 | Spain |
| HM596459 | *Apodemus sylvaticus* | Gil et al. 2010 | Spain |
| HM596460 | *Apodemus sylvaticus* | Gil et al. 2010 | Spain |
| HM596461 | *Apodemus sylvaticus* | Gil et al. 2010 | Spain |
| HM596463 | *Apodemus sylvaticus* | Gil et al. 2010 | Spain |
| HM596464 | *Apodemus sylvaticus* | Gil et al. 2010 | Spain |
| HM596468 | *Mus spretus* | Gil et al. 2010 | Spain |
| HM596469 | *Mus musculus* | Gil et al. 2010 | Spain |
| JF500515 | *Niviventer coxingi* | Lin et al. 2012 | Taiwan |
| JQ694004 | *Apodemus agrarius* | Hildebrand et al. 2013 | Poland |
| JQ694005 | *Apodemus agrarius* | Hildebrand et al. 2013 | Poland |
