# Supplementary material for: Contrasting Patterns in Mammal–Bacteria Coevolution: Bartonella and Leptospira in Bats and Rodents
Source: PLoS Negl Trop Dis. 2014 Mar 20;8(3):e2738. doi: 10.1371/journal.pntd.0002738 (PMC3961187; doi:10.1371/journal.pntd.0002738)
Supplement: Table S3 — 16S GenBank accession numbers for studied Leptospira sequences in bat hosts. (DOCX) [file pntd.0002738.s004.docx]

**Table S3.** 16S GenBank accession numbers for studied *Leptospira* sequences in bat hosts

| GenBank Accession | Host Species | Paper | Country |
| --- | --- | --- | --- |
| AY995712 | *Uroderma bilobatum* | Matthias et al. 2005 | Peru |
| AY995713 | *Sturnira lilium* | Matthias et al. 2005 | Peru |
| AY995714 | *Sturnira tildae* | Matthias et al. 2005 | Peru |
| AY995715 | *Desmodus rotundus* | Matthias et al. 2005 | Peru |
| AY995716 | *Carollia perspicillata* | Matthias et al. 2005 | Peru |
| AY995717 | *Lonchophylla thomasi* | Matthias et al. 2005 | Peru |
| AY995718 | *Artibeus planirostris* | Matthias et al. 2005 | Peru |
| AY995719 | *Artibeus planirostris* | Matthias et al. 2005 | Peru |
| AY995720 | *Rhinophylla pumilio* | Matthias et al. 2005 | Peru |
| AY995721 | *Glossophaga soricina* | Matthias et al. 2005 | Peru |
| AY995722 | *Mimon crenulatum* | Matthias et al. 2005 | Peru |
| AY995723 | *Myotis riparius* | Matthias et al. 2005 | Peru |
| AY995724 | *Lonchophylla thomasi* | Matthias et al. 2005 | Peru |
| AY995725 | *Uroderma bilobatum* | Matthias et al. 2005 | Peru |
| AY995726 | *Glossophaga soricina* | Matthias et al. 2005 | Peru |
| AY995727 | *Artibeus obscurus* | Matthias et al. 2005 | Peru |
| AY995728 | *Rhinophylla pumilio* | Matthias et al. 2005 | Peru |
| AY995729 | *Promops nasutus** | Matthias et al. 2005 | Peru |
| AY995730 | *Phyllostomus hastatus* | Matthias et al. 2005 | Peru |
| JQ288729 | *Otomops madagascariensis* | Lagadec et al. 2012 | Madagascar |
| JQ288730 | *Miniopterus mahafaliensis* | Lagadec et al. 2012 | Madagascar |
| JQ288731 | *Triaenops menamena** | Lagadec et al. 2012 | Madagascar |
| JQ288732 | *Rousettus obliviosus* | Lagadec et al. 2012 | Union of the Comoros |
| JQ288733 | *Rousettus obliviosus* | Lagadec et al. 2012 | Union of the Comoros |
| JQ288734 | *Miniopterus griveaudi* | Lagadec et al. 2012 | Union of the Comoros |
| JQ302791 | *Myotis goudoti* | Lagadec et al. 2012 | Madagascar |

*host species does not have cytochrome b sequence in GenBank, used most closely related species for host-parasite associations
