# Supplementary material for: Contrasting Patterns in Mammal–Bacteria Coevolution: Bartonella and Leptospira in Bats and Rodents
Source: PLoS Negl Trop Dis. 2014 Mar 20;8(3):e2738. doi: 10.1371/journal.pntd.0002738 (PMC3961187; doi:10.1371/journal.pntd.0002738)
Supplement: Table S4 — Cytochrome b GenBank accession numbers of bat species host to studied bacteria. (DOCX) [file pntd.0002738.s005.docx]

**Table S4.** Cytochrome b GenBank accession numbers of bat species host to studied bacteria

| Species | GenBank Accession | Host to *Leptospira* | Host to *Bartonella* |
| --- | --- | --- | --- |
| *Apodemus agrarius* | AB032851 |  | x |
| *Apodemus peninsulae* | AB032850 |  | x |
| *Artibeus jamaicensis* | U66503 |  | x |
| *Artibeus obscurus* | AF423079 | x | x |
| *Artibeus planirostris* | U66508 | x | x |
| *Artibeus toltecus* | U66515 |  | x |
| *Brachyphylla cavernarum* | AY572377 |  | x |
| *Carollia perspicalata* | FJ589655 | x | x |
| *Clethrionomys rufocanus* | AB031553 |  | x |
| *Coleura afra* | HQ693720 |  | x |
| *Desmodus rotundus* | DQ077398 | x | x |
| *Eidolon helvum* | JN398200 |  | x |
| *Glossophaga soricina* | FJ392505 | x | x |
| *Hipposideros armiger** | JX465363 |  | x |
| *Lonchophylla thomasi* | AF187034 | x |  |
| *Micronycteris microtis* | AY380756 |  | x |
| *Microtus fortis* | FJ986307 |  | x |
| *Mimon crenulatum* | FJ155478 | x |  |
| *Miniopterus griveaudi* | FJ232793 | x |  |
| *Miniopterus mahafaliensis* | FJ383160 | x |  |
| *Miniopterus schreibersii* | AB444719 |  | x |
| *Monophyllus redmani* | AF382888 |  | x |
| *Myotis daubentoni* | AB106589 |  | x |
| *Myotis goudoti* | GU116764 | x |  |
| *Myotis mystacinus* | AB106605 |  | x |
| *Myotis riparius* | AF376866 | x |  |
| *Nyctalus noctula* | JX570902 |  | x |
| *Otomops madagascariensis* | EF216372 | x |  |
| *Phyllostomus hastatus** | FJ155479 | x | x |
| *Pipistrellus abramus* | AB085739 |  | x |
| *Promops centralis** | L19732 | x |  |
| *Pteronotus davyi* | AF338669 |  | x |
| *Rhinophylla pumilio* | AF187029 | x |  |
| *Rousettus aegyptiacus* | AB085740 | x |  |
| *Rousettus obliviosus* | GU228728 |  | x |
| *Sturnira lilium* | AF187035 | x | x |
| *Sturnira tildae* | KC753887 | x |  |
| *Triaenops persicus** | EU798758 |  | x |
| *Uroderma bilobatum* | AY169900 | x |  |
| *Vampyressa bidens* | AY157044 |  | x |
