# Supplementary material for: Contrasting Patterns in Mammal–Bacteria Coevolution: Bartonella and Leptospira in Bats and Rodents
Source: PLoS Negl Trop Dis. 2014 Mar 20;8(3):e2738. doi: 10.1371/journal.pntd.0002738 (PMC3961187; doi:10.1371/journal.pntd.0002738)
Supplement: Table S5 — Cytochrome b GenBank accession numbers of rodent species host to studied Bartonella. (DOCX) [file pntd.0002738.s006.docx]

**Table S5.** Cytochrome b GenBank accession numbers of rodent species host to studied *Bartonella*

| Species | GenBank Accession |
| --- | --- |
| *Acomys cahirinus* | AJ233953 |
| *Acomys russatus* | FJ415485 |
| *Aethomys namaquensis* | AF141215 |
| *Apodemus agrarius* | AB303226 |
| *Apodemus flavicollis* | AB032853 |
| *Apodemus peninsulae* | AB073811 |
| *Apodemus sylvaticus* | AB033695 |
| *Callosciurus notatus* | AB499913 |
| *Clethrionomys rufocanus* | AB031580 |
| *Crocidura russula* | AY332699 |
| *Dryomys nitedula* | AJ225116 |
| *Eutamias sibiricus* | AF147667 |
| *Gerbillus pyramidum* | JN652813 |
| *Glaucomys volans* | AB030261 |
| *Jaculus jaculus* | JX885206 |
| *Jaculus orientalis* | JN652664 |
| *Mastomys natalensis* | JQ735706 |
| *Microtus agrestis* | AY332712 |
| *Microtus fortis* | FJ986308 |
| *Mus cervicolor* | AB125766 |
| *Mus domesticus* | AB125774 |
| *Mus spretus* | AB033700 |
| *Myodes rutilus* | JX885766 |
| *Neomys fodiens* | AB175071 |
| *Pachyuromys duprasi* | AJ851274 |
| *Peromyscus maniculatus* | JF489123 |
| *Psammomys obesus* | AJ851275 |
| *Rattus exulans* | JX534034 |
| *Rattus rattus* | AB033702 |
| *Niviventer coxingi*  *Rattus norvegicus* | EF053026  AB033713 |
| *Rattus tanezumi* | AB211043 |
| *Rhabdomys pumilio* | AF533116 |
| *Sekeetamys calurus* | AJ851276 |
| *Sorex coronatus* | AJ000419 |
| *Spermophilus beecheyi* | AF157919 |
| *Spermophilus dauricus* | AF157899 |
| *Suncus murinus* | AB033610 |
| *Talpa europaea* | AB037601 |
| *Tamias minimus* | AF147650 |
| *Tamiasciurus hudsonicus* | AF147643 |
| *Tatera leucogaster* | AJ851260 |
| *Urocitellus columbianus* | JQ679123 |
| *Urocitellus richardsonii* | S73150 |
