# Supplementary material for: Contrasting Patterns in Mammal–Bacteria Coevolution: Bartonella and Leptospira in Bats and Rodents
Source: PLoS Negl Trop Dis. 2014 Mar 20;8(3):e2738. doi: 10.1371/journal.pntd.0002738 (PMC3961187; doi:10.1371/journal.pntd.0002738)
Supplement: Table S6 — Results from event-based cophylogeny Jane, using different cost-settings. (DOCX) [file pntd.0002738.s007.docx]

**Table S6.** Results from event-based cophylogeny Jane, using different cost-settings

| Cost scheme | Cospeciations | Duplications | Duplications and host switches | Losses | Failures to diverge | Total Cost |
| --- | --- | --- | --- | --- | --- | --- |
| *Bats-Bartonella* |  |  |  |  |  |  |
| 0, 1, 2, 1, 1 | 10 | 21 | 24 | 4 | 0 | 73 |
| 10, 1, 2, 1, 1 | 0 | 23 | 32 | 0 | 0 | 87 |
| 0, 1, 10, 1, 1 | 17 | 38 | 0 | 116 | 0 | 154 |
| 0, 1, 2, 10, 1 | 6 | 21 | 28 | 0 | 0 | 77 |
| 0, 1, 2, 1, 10 | 10 | 21 | 24 | 4 | 0 | 73 |
| *New World* |  |  |  |  |  |  |
| 0, 1, 2, 1, 1 | 5 | 14 | 18 | 3 | 0 | 53 |
| 10, 1, 2, 1, 1 | 0 | 16 | 21 | 0 | 0 | 58 |
| 0, 1, 10, 1, 1 | 10 | 25 | 2 | 62 | 0 | 107 |
| 0, 1, 2, 10, 1 | 3 | 13 | 21 | 0 | 0 | 55 |
| 0, 1, 2, 1, 10 | 5 | 14 | 18 | 3 | 0 | 53 |
| *Old World* |  |  |  |  |  |  |
| 0, 1, 2, 1, 1 | 6 | 6 | 5 | 1 | 0 | 17 |
| 10, 1, 2, 1, 1 | 0 | 8 | 9 | 0 | 0 | 26 |
| 0, 1, 10, 1, 1 | 6 | 11 | 0 | 20 | 0 | 31 |
| 0, 1, 2, 10, 1 | 5 | 6 | 6 | 0 | 0 | 18 |
| 0, 1, 2, 1, 10 | 6 | 6 | 5 | 1 | 0 | 17 |
|  |  |  |  |  |  |  |
| *Bats-Leptospira* |  |  |  |  |  |  |
| 0, 1, 2, 1, 1 | 6 | 0 | 19 | 3 | 0 | 41 |
| 10, 1, 2, 1, 1 | 0 | 2 | 23 | 0 | 0 | 48 |
| 0, 1, 10, 1, 1 | 11 | 14 | 0 | 99 | 0 | 113 |
| 0, 1, 2, 10, 1 | 3 | 1 | 21 | 0 | 0 | 43 |
| 0, 1, 2, 1, 10 | 6 | 0 | 19 | 3 | 0 | 41 |
| *New World* |  |  |  |  |  |  |
| 0, 1, 2, 1, 1 | 5 | 1 | 12 | 2 | 0 | 27 |
| 10, 1, 2, 1, 1 | 0 | 2 | 16 | 0 | 0 | 34 |
| 0, 1, 10, 1, 1 | 8 | 9 | 1 | 45 | 0 | 64 |
| 0, 1, 2, 10, 1 | 3 | 1 | 14 | 0 | 0 | 29 |
| 0, 1, 2, 1, 10 | 5 | 1 | 12 | 2 | 0 | 27 |
| *Old World* |  |  |  |  |  |  |
| 0, 1, 2, 1, 1 | 2 | 0 | 4 | 1 | 0 | 9 |
| 10, 1, 2, 1, 1 | 0 | 1 | 5 | 0 | 0 | 11 |
| 0, 1, 10, 1, 1 | 2 | 4 | 0 | 15 | 0 | 19 |
| 0, 1, 2, 10, 1 | 1 | 0 | 5 | 0 | 0 | 10 |
| 0, 1, 2, 1, 10 | 2 | 0 | 4 | 1 | 0 | 9 |
|  |  |  |  |  |  |  |
| *Rodents-Bartonella* | |  |  |  |  |  |
| 0, 1, 2, 1, 1 | 17 | 77 | 34 | 123 | 11 | 279 |
| 10, 1, 2, 1, 1 | 0 | 77 | 51 | 118 | 11 | 308 |
| 0, 1, 10, 1, 1 | 27 | 99 | 2 | 251 | 11 | 381 |
| 0, 1, 2, 10, 1 | 9 | 76 | 43 | 114 | 11 | 1313 |
| 0, 1, 2, 1, 10 | 17 | 77 | 34 | 122 | 11 | 377 |
| *New World* |  |  |  |  |  |  |
| 0, 1, 2, 1, 1 | 1 | 15 | 3 | 0 | 0 | 21 |
| 10, 1, 2, 1, 1 | 0 | 16 | 3 | 0 | 0 | 22 |
| 0, 1, 10, 1, 1 | 2 | 17 | 0 | 9 | 0 | 26 |
| 0, 1, 2, 10, 1 | 1 | 15 | 3 | 0 | 0 | 21 |
| 0, 1, 2, 1, 10 | 1 | 15 | 3 | 0 | 0 | 21 |
| *Old World* |  |  |  |  |  |  |
| 0, 1, 2, 1, 1 | 17 | 54 | 37 | 75 | 11 | 214 |
| 10, 1, 2, 1, 1 | 0 | 56 | 52 | 75 | 11 | 246 |
| 0, 1, 10, 1, 1 | 27 | 77 | 4 | 202 | 11 | 330 |
| 0, 1, 2, 10, 1 | 11 | 54 | 43 | 69 | 11 | 841 |
| 0, 1, 2, 1, 10 | 17 | 54 | 37 | 75 | 11 | 313 |
